# A Case of Small-Pox after Vaccination

**Published:** 1805-07-01

**Authors:** 

**Affiliations:** Surgeon of the Lock Hospital, &c.


### A Case *of* Small-pox *after* Vaccination; *communicated by* Mr. Blair, *Surgeon of the Lock Hospital, &c.*

On May 7, 1803, I inoculated a child, named Alice Gorthorpe, with the vaccine fluid. The disease made its usual progress, and left a cicatrix on each arm, as may be seen at present. On the eighth day after inoculation, I took matter from this child, and vaccinated several others.

On Monday, June 3, 1805, the mother of the child, residing at No. 25, Tower Street, Seven Dials, came to my house, and shewed me the patient covered with a distinct variolous eruption, small in size, but fairly maturated. I sent her immediately to Dr. Adams, (who succeeded Dr. Woodville at the Inoculation Hospital) to prevent my giving an erroneous opinion respecting her case; he agreed with me that it was certainly the small-pox; and that this instance must be added to the few others which have occurred, of the variolous infection taking place subsequent to complete vaccination.

I have only to add, that three years ago I vaccinated another child of the same parent, and a third on the 21st of May last; both of which, with nine more who had been inoculated in the same family, escaped the small-pox on on this occasion; and, what is deserving of notice, the infant whom I vaccinated on the 21st of last month (Thomas Gorthorpe) had slept in the same bed with his sister during the whole progress of her recent disease.

*Great Russel Street, Bloomsbury, June 6, 1805.*

